# Maize kernel metabolome involved in resistance to fusarium ear rot and fumonisin contamination

**DOI:** 10.3389/fpls.2023.1160092

**Published:** 2023-07-19

**Authors:** Ana Cao, Noemi Gesteiro, Rogelio Santiago, Rosa Ana Malvar, Ana Butrón

**Affiliations:** ^1^ Misión Biológica de Galicia (CSIC), Pontevedra, Spain; ^2^ Agrobiología Ambiental, Calidad de Suelos y Plantas (UVIGO), Unidad Asociada a la MBG (CSIC), Pontevedra, Spain

**Keywords:** *Zea mays* L., maize, *Fusarium verticillioides*, fumonisins, untarget metabolomics, resistance, fusarium ear rot

## Abstract

*Fusarium verticillioides* poses a threat to worldwide maize production due to its ability to infect maize kernel and synthesize fumonisins that can be accumulated above safety levels for humans and animals. Maize breeding has been proposed as key tool to decrease kernel contamination with fumonisins, but metabolic studies complementary to genomic approaches are necessary to disclose the complexity of maize resistance. An untargeted metabolomic study was proposed using inbreds genetically related but with contrasting levels of resistance in order to uncover pathways implicated in resistance to Fusarium ear rot (FER) and fumonisin contamination in the maize kernel and to look for possible biomarkers. Metabolite determinations were performed in kernels collected at 3 and 10 days after inoculation with *F. verticillioides* (dat). Discriminant metabolites between resistant and susceptible RILs were rather found at 10 than 3 dat, although metabolite differences at later stages of colonization could be driven by subtle variations at earlier stages of infection. Within this context, differences for membrane lipid homeostasis, methionine metabolism, and indolacetic acid conjugation seemed highly relevant to distinguish between resistant and susceptible inbreds, confirming the polygenic nature of resistance to FER and fumonisin contamination in the maize kernels. Nevertheless, some specific metabolites such as the polyamine spermidine and/or the alkaloid isoquinoline seemed to be promising indirect selection traits to improve resistance to FER and reduce fumonisin accumulation. Therefore, *in vitro* and *in vivo* experiments will be necessary to validate the inhibitory effects of these compounds on fumonisins biosynthesis.

## Introduction

*Fusarium verticillioides* poses a threat to worldwide maize production because maize kernels infected with this fungus can accumulate fumonisins above safety levels for animal and human health. Fumonisin accumulation is mainly determined by environmental conditions, but there is wide genetic variability in maize for resistance to fumonisin accumulation that can be used to generate maize varieties with reduced fumonisin content (Eller et al, 2008; Santiago et al, 2015). However, breeding gains are hindered by the complex inheritance of kernel resistance to fumonisin accumulation due to the highly polygenic nature of the trait and the large effect of the genotype x environment interaction (Santiago et al, 2020). In such scenario, the predictive power of genetic data can be significantly improved when combined with metabolic measurements, because metabolites are the result of both genetic and environmental factors and, as such, provide great potential to bridge knowledge between genotype and phenotype (Gartner et al, 2009; Schrimpe-Rutledge et al, 2016). In this sense, untargeted metabolomics offers a discovery and hypothesis-generating approach to provide new biomarkers to be used in plant breeding, as well as valuable insights into the pathways used by maize to limit kernel contamination with fumonisins (Gauthier et al, 2015; Ribbenstedt et al, 2018). In addition, these metabolic studies could complement and/or corroborate results previously obtained by using transcriptomic and proteomic approaches (Lanubile et al., 2010; Lanubile et al., 2012; Campos-Bermudez et al., 2013; Yuan et al., 2013; Lanubile et al., 2014; Wang et al., 2016; Lanubile et al., 2017; Cao et al., 2022).

Campos-Bermudez et al. (2013) studied the transcriptomic and metabolomic changes associated to kernel infection by *F. verticillioides* in mature kernels of two unrelated inbreds with different performance against infection by *F. verticillioides.* These authors found no significant changes in transcriptional and metabolomic profiles between resistant and susceptible inbreds, and they suggested that a constitutive defense mechanism may confer the resistant inbred an advantage against *F. verticillioides* infection. Righetti et al. (2019) investigated differences in mature kernel metabolic profiles among three maize commercial hybrids under natural inoculation at open-field conditions and concluded that the maize lipid signature was strongly involved in the maize-*F. verticillioides* interaction and in the modulation of fumonisin accumulation; maize lipidome signature being genotype dependent. However, as metabolic differences among unrelated genotypes with contrasting values for resistance to fumonisin contamination could be attributed to genes related to resistance but also to many other genes these genotypes differ for, metabolomic studies aimed at identifying relevant biomarkers of genotype resistance should be performed with genotypes genetically related to avoid the possible biased caused by the genetic background. Ciasca et al. (2020) studied the metabolomics changes of two maize recombinant inbred lines with contrasting phenotypes obtained from the same cross, but they sampled germinated kernels instead of intact kernels. In the current study, we propose to compare the metabolomes of recombinant inbred lines (RILs) derived from the same cross but differing for resistance to kernel contamination with fumonisins to eliminate as much as possible the influence of genetic background on metabolic differences. Four different RILs were included in each category, resistant and susceptible, and would be identical in one or more genomic regions implicated in resistance but arbitrary in all unlinked regions (Michelmore et al, 1991). Specifically, this study was focused on maize metabolomics of immature kernels since genotype-driven specific resistance factors could act at the beginning of maize colonization by the fungus and would be no longer detectable at the harvest stage (Righetti et al, 2021). In this sense, authors have reported that fumonisin production can be initiated during late-milk stage (approximately 18 days after pollination), but plant resistance mechanisms appear to became relevant for reducing fumonisin accumulation between 22 and 29 days after pollination (Picot et al, 2011; Maschietto et al, 2015). Therefore, in order to find compounds in developing maize kernels that contribute most to the resistance to fumonisin accumulation, kernels should be harvested at the milk-dough stage (Atanasova-Penichon et al, 2014). Taking all this into account the main objectives of this study were (i) to uncover metabolic pathways implicated in resistance to FER and fumonisin contamination in the maize kernel and (ii) to identify possible biomarkers to be used in future breeding programs.

## Materials and methods

### Experiment setup and sample collection

Eight RILs of maize were selected from a set of 144 RILs derived from the cross between the European flint inbred line EP42 (susceptible to FER and kernel contamination with fumonisins) and the American dent inbred line A637 (resistant), and previously genotyped and phenotyped for FER and kernel fumonisin content under inoculation with *F. verticillioides* (Santiago et al, 2013; Samayoa et al, 2014; Cao et al, 2022). The four RILs with the lowest values (resistant) for fumonisin content (10-15 µg/g) and FER (~ 2 in a visual scale from 1 to 7) along with the four RILs with the highest values (susceptible) for fumonisin content (55-75 µg/g) and FER (~ 4) were selected based on previous evaluations (**Supplementary Table 1**) (Cao et al., 2022). In 2018, 15 seeds from each RIL were sown in a single row of 3.5 m; distance between adjacent rows being 0.8 m. RILs were arranged in four pairs, each pair formed with one resistant and one susceptible RIL. RILs within each pair were planted in adjacent rows in order to minimize the contribution of field heterogeneity to differential metabolite content between resistant and susceptible inbreds. We self-crossed at least six plants per RIL, and 15 days later the main ear of each plant was inoculated, using a kernel inoculation technique (Cao et al., 2014), with a spore suspension of *F. verticillioides* as previously described (Cao et al, 2022). Ears were individually collected at 3 or 10 days after inoculation treatment (dat) (18 and 25 days after pollination, respectively). Immediately, undamaged immature kernels around the inoculation point were carefully collected into closed cap tubes kept in liquid nitrogen and stored at – 80 °C until lyophilization. Three biological replicates (ears) were obtained for each RIL and sampling date, except for one susceptible RIL at the 3-day sampling date for which no ears were obtained.

### Metabolite extraction

Lyophilized kernels were ground in a mortar and 20 mg per sample were extracted twice with 1 ml of 75% methanol in acetate buffer, mixed in a vortex, sonicated (ultrasonic bath 30 Hz for 5 min) and centrifuged (20,000 g for 10 min). The supernatants were combined and filtered through a 0.22 µm PTFE membrane filter to an Eppendorf tube and an aliquot was transferred to a certified vial. Samples were stored at 4° C until analysis. For MS/MS analysis sample replicates pools were prepared in separate vials.

### Untargeted liquid chromatography–mass spectrometry

The untargeted metabolomic analysis was conducted by ultra-high-performance liquid chromatography (UHPLC) (Ultimate 3000 LC; Thermo Scientific) coupled to a quadrupole-time-of-flight mass spectrometer (QTOF-MS) equipped with an electrospray ionization source (ESI) (Bruker Compact; Bruker Daltonics). Samples (5 µl injection volume) were separated in an Intensity Solo 2 C18 column (1.7 µm, 2.1× 100 mm; Bruker Daltonics) at 35°C. A binary solvent system consisted of 0.1% of formic acid on water (solvent A) and acetonitrile (solvent B) with a 0.4 ml/min flow rate was used with the following gradient conditions: 0 min, 3% B; 4 min, 3% B; 16 min, 25% B; 25min, 80% B; 30 min, 100% B; 32 min, 100% B; return to initial conditions at 33 min (3% B) and maintain until 36 min.

The MS acquisition was performed in both negative and positive ionization modes for full scan and auto MS/MS, in a mass scan range of m/z 100-1200. Specific conditions used were: gas flow 9 L/min, nebulizer pressure 38 psi, dry gas 9 L/min, and dry temperature 220°C; capillary and end plate offset were set to 4500 and 500 V, respectively. MS/MS analysis was performed by using different collision energy ramps to cover a range from 15 to 50 eV. The instrument was calibrated externally with a solution of 1mM sodium formate/acetate in 2-propanol:water 50:50 with 0.2% formic acid directly infused to the source. The calibration solution was injected at the beginning of each run and all the spectra were calibrated prior to statistical analysis.

### Data processing and statistical analyses

The UHPLC-MS and MS/MS raw data were processed using the MetaboScape 4.0 software (Bruker Daltonics) and the algorithm T–Rex 3D for peak detection and alignment in a retention time (Rt) range from 0.5 to 30 min. Data obtained from positive and negative ionization modes were combined and system contaminants were manually removed (**Supplemental Tables 2, 3**). The web server MetaboAnalyst 5.0 (Chong et al, 2019) was used for further data filtering and statistical analyses. Variables with more than 50% missing values were removed and all missing values were replaced with a low value (1/5 of the minimum positive value of each variable). Then, the interquantile range filter was used to remove uninformative variables with a near-constant values throughout the dataset and a Pareto scaling was applied for adjusting for the disparities in fold differences between the metabolites. Within each sampling date, Orthogonal Projections to Latent Structures Discriminant Analysis (OPLS‐DA) was carried out to investigate and visualize the pattern of metabolite changes between the resistant and susceptible RILs. OPLS-DA disentangle group-predictive and group-unrelated variation in the measured data and provides a model in which the variables with the largest discriminatory power between groups are determined (Bylesjo et al, 2006). The OPLS‐DA model was evaluated through cross‐validation and the R2Y (estimation of the goodness of fit of the model) and Q2 (qualitative measure of the predictive ability of the model) statistics were used for quality assessment. We performed 1000-run permutations to test the possibility of obtaining those values for the goodness of fit and predictability by chance. In addition, the fold change ratio (FC) and *p*-value of the t-test for each peak between resistant and susceptible inbreds were calculated. For each sampling date, features with a variable importance in projection (VIP) score >1 in the OPLS‐DA model, |Log_2_FC| > 0.6 and *p* value < 0.05 were considered as significant and those with FDR < 0.10, VIP score >1, and |Log_2_FC| > 0.6 as highly significant.

### Metabolite annotation

Significant metabolite features were annotated based on the accurate mass, molecular formula and fragmentation spectrum when available. MetaboScape 4.0 software and the bioinformatic tool SIRIUS 4 (version 4.9.12) (Duhrkop et al, 2019) were used for molecular formulas calculations, and the CSi : FingerID tool (Duhrkop et al, 2015) in SIRIUS 4 and the spectral library MS-DIAL (Tsugawa et al, 2020) for molecular structures and MS/MS experimental spectra comparisons in metabolomic databases. Putative annotations were performed using publicly available databases as Pubchem (https://pubchem.ncbi.nlm.nih.gov ), Lipid Maps (https://www.lipidmaps.org), KEGG (https://www.genome.jp), ChEBI, (https://www.ebi.ac.uk/chebi), MoNA (https://mona.fiehnlab.ucdavis.edu), PlantCyc (https://plantcyc.org) (all accesed between February and October 2022), and consulting literature references.

### Pathway analyses

For addressing the metabolic pathways and network-level changes in the resistant vs susceptible RILs, we performed a joint analysis with mummichog and gene set enrichment analysis (GSEA) using the Functional Analysis module of MetaboAnalyst (Pang et al, 2021). These computational algorithms predict functional activity from mass spectrometry data without *a priori* identification of metabolites by leveraging the collective power of metabolic pathways and networks. The mummichog algorithm infers pathway activities using an over-representation analysis method to evaluate pathway-level enrichment based on significant features (with *p*-values above a *p*-value cutoff) from a ranked list of MS peaks identified by untargeted metabolomics (Li et al. (2013). The GSEA method (Subramanian et al, 2005) can extract biological meaning from a ranked feature list (based on *t s*cores) without using a significance cutoff. The setup parameters used to perform functional analysis were: the library selected was the KEGG pathway library for *Oryza sativa japonica*; a mass tolerance of 5 ppm was stablished for putative annotation; and mummichog default cutoffs for *p*-values were used (0.1 and 0.01 for metabolite data at 3 and 10 dat, respectively).

Finally, we used the Pathview platform (Luo and Brouwer, 2013; Luo et al, 2017) to integrate and visualize previous transcriptomic data (Cao et al, 2022) and the current untargeted metabolomic data. Target pathways for visualization were: phenylpropanoid biosynthesis, and glutathione and glycerophospholipid metabolisms.

## Results

**Figure 1:**
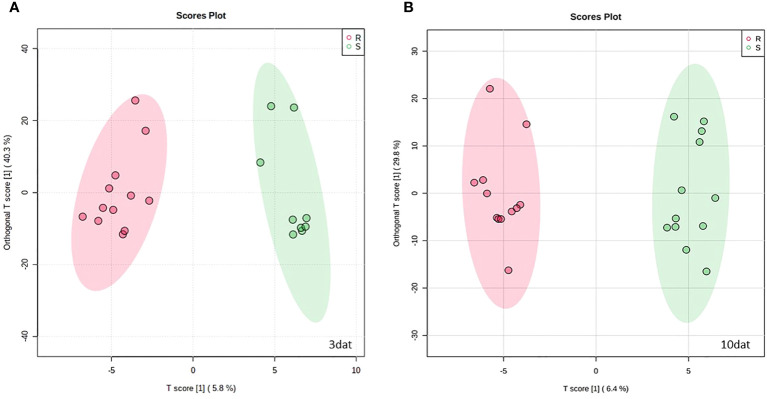
Biplot representation of the scores of resistant (R), in red, and susceptible (S), in green, recombinant inbred lines of maize for predictive and first orthogonal components of supervised least square and orthogonal projections to latent structures discriminant analysis (OPLS-DA) for metabolomic data acquired at **(A)** 3 and **(B)** 10 days after inoculation (dat) with *F. verticillioides*.

738 and 609 features were identified in kernel samples taken three and 10 days, respectively, after inoculation with *Fusarium verticillioides* (**Supplementary Tables 2, 3**). In order to identify the metabolomic differences between resistant and susceptible maize RILs, we first performed an OPLS-DA of the metabolomic data. The OPLS-DA model for kernel samples harvested at 3 dat explained 63% of metabolite variability (R2X); 57.3% of variability was structured in three orthogonal components meanwhile only the 5.8% of metabolite variation was predictive and assisted in differentiating resistant from susceptible inbreds (**Figure 1** and **Supplemental Figure 1**). The model had an overall goodness of fit (R2Y) of 0.961 and an overall cross-validation coefficient (Q2) of 0.504; the probabilities of obtaining those values by chance being 0.083 for R2Y and 0.007 for Q2, respectively.

The OPLS-DA model for kernel samples collected at 10 dat comprised one predictive and three orthogonal components; predictive metabolite variability being the 6.4% of total metabolite variability (**Figure 1** and **Supplemental Figure 1**). R2Y was 0.975 (*p* < 0.001) and Q2 0.714 (*p* < 0.001). The overall metabolite variability explained by the model was 55.5%, most variability being structured but uncorrelated to the differentiation between resistant and susceptible RILs.

**Figure 2:**
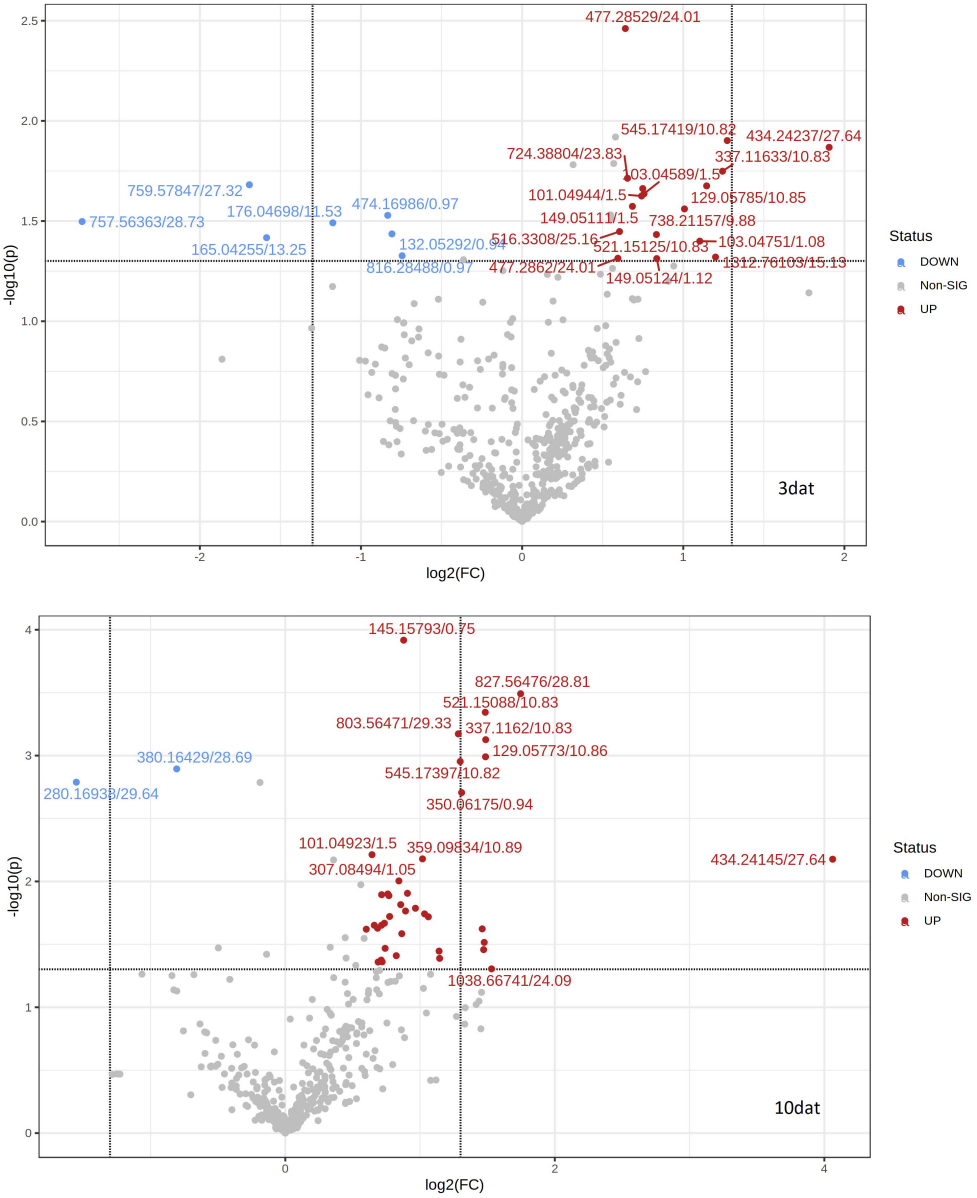
Volcano plot of differentially accumulated features in kernels collected 3 (3dat) and 10 (10dat) days after inoculation with F. verticillioides in resistant and susceptible RILs. Up-regulated (UP), marked in red, and down-regulated (DOWN), marked in blue, features were more and less, respectively, accumulated in resistant versus susceptible RILs. Grey dots corresponded to features not differentially accumulated (Non-SIG). Labels of features were composed of the neutral mass/retention time.

**Figure 2** shows the metabolite features from kernel samples harvested 3 and 10 dat with FC higher than 1.5 or lower than 0.7 (|Log_2_FC | > 0.6) and *p* values less than 0.05 for t-test between resistant and susceptible RILs. Discriminant features with a VIP score > 1 in the OPLS-DA model and that fulfilled the above mentioned FC and t-test criteria were considered as differentially accumulated metabolites in the resistant vs susceptible RILs (**Tables 1**, **2**). However, only 10 metabolites out of them were high-significant differentially (FDR < 0.10, *p* value < 0.002) accumulated at 10 dat and no metabolite at 3 dat in contrast with most metabolomic studies in which differentially accumulated features between treatments on a single genotype are studied. In the current study, the genetically heterogeneous composition of each bulk could be behind the reduced number of features differentially accumulated between resistant and susceptible RILs. However, detected features are expected to play significant roles in resistance as they would be regulated by genomic regions shared by all resistant or susceptible RILs.

**Table 1:** LC-MS/MS data, tentative annotation, fold changes, *p* values and VIP scores for differentially accumulated metabolites between resistant and susceptible maize RILs at 3 days after inoculation with *Fusarium verticillioides*.

RT (min)	Ionization	m/z	Neutral mass	MS/MS fragments (m/z)	Molecular formula	Error (|Δ m/z| ppm)	Tentative compound	FC	log_2_FC	*p*	VIP
0.94	[M-H]^-^	131.0456	132.0529	n.d.	C_4_H_8_N_2_O_3_	4.37	Asparagine	0.57	-0.81	0.0366	2.21
0.97	[M-H]^-^	815.2776	816.2849	n.d.				0.60	-0.74	0.0471	2.08
0.97	[M-H]^-^	473.1626	474.1699	341.107, 293.107, 179.054, 161.042, 131.045, 113.035	C_16_H_30_N_2_O_14_	0.33	Asparagine di-hexoside	0.56	-0.83	0.0296	2.32
1.08	[M+H]^+^	104.0547	103.0475	n.d.				2.15	1.10	0.0398	1.70
1.12	[M+H]^+^	150.0585	149.0512	104.052, 84.047, 74.023, 61.011, 56.050	C_5_H_11_NO_2_S	0.92	Methionine	1.78	0.84	0.0487	1.67
1.50	[M+H]^+^	150.0583	149.0511	104.052, 84.047, 74.023, 61.011, 56.050	C_5_H_11_NO_2_S	0.44	Methionine	1.61	0.68	0.0267	1.81
1.50	[M+H]^+^	133.0320	132.0248	61.012. 56.051	C_5_H_8_O_2_S	1.86	Methionine derivative	1.68	0.75	0.0218	1.78
1.50	[M+H]^+^	104.0531	103.0459	n.d.	C_4_H_9_NS	2.60	Methionine derivative	1.69	0.76	0.0231	1.77
1.50	[M+H]^+^	102.0551	101.0494	n.d.	C_4_H_7_NO_2_	1.76	Methionine derivative	1.67	0.74	0.0237	1.76
9.88	[2M-H]^-^	737.2042	738.2116	368.097, 206.045, 162.053	C_16_H_19_NO_9_	1.21	Hydroxy-oxindole-3-acetyl-hexoside	2.01	1.01	0.0275	2.14
10.82	[M+HCOOH-H]^−^	544.1669	499.1687	498.154, 341.107, 323.097, 203.055, 179.055, 174.055, 161.044, 143.034, 130.065, 119.034, 113.023	C_22_H_29_NO_12_	0.24	Indole-3-acetyl-*myo*-inositol hexoside	2.42	1.27	0.0125	2.23
10.83	[M+Na]^+^	522.1585	499.1693	348.098, 290.066, 203.046, 177.067, 130.066	C_22_H_29_NO_12_	0.66	Indole-3-acetyl-*myo*-inositol hexoside	1.78	0.83	0.0369	1.92
10.83	[M+H]^+^	338.1236	337.1163	176.071, 130.065, 109.029, 81.034, 57.033	C_16_H_19_NO_7_	0.43	Indole-3-acetyl-hexoside	2.37	1.24	0.0178	2.18
10.85	[M+H]^+^	130.0651	129.0579	105.048, 77.039, 53.039	C_9_H_7_N	1.04	Isoquinoline	2.21	1.15	0.0211	2.13
11.53	[M-H_2_O+H]^+^	177.0543	194.0574	117.035, 105.043, 97.011, 89.042, 78.042, 53.041	C_10_H_10_O_4_	1.55	Ferulic acid	0.44	-1.17	0.0323	2.02
13.25	[M-H]^-^	164.0353	165.0426	149.009, 121.015, 96.962	C_8_H_7_NO_3_	0.28	MBOA	0.33	-1.59	0.0383	2.20
15.13	[M+H+H_2_]^3+^	438.5943	1312.7610	n.d.				2.30	1.20	0.0478	1.51
23.83	[M+HCOOH-H]^−^	723.3808	724.3880	677.368, 415.145, 397.134, 379.122, 323.097, 305.088, 279.232, 235.082, 179.056, 119.035	C_33_H_58_O_14_	0.72	DGMG 18:2	1.57	0.65	0.0193	1.91
24.01	[M-H]^-^	476.2780	477.2853	279.232, 214.048, 196.037, 140.012	C_23_H_44_NO_7_P	0.52	LysoPE 18:2	1.56	0.64	0.0035	2.21
24.01	[M+H]^+^	478.2936	477.2862	337.267, 306274, 255.149, 173.017, 121,098, 95.084, 62.061	C_23_H_44_NO_7_P	1.64	LysoPE 18:2	1.51	0.59	0.0485	1.83
25.16	[M+Na]+	539.3202	516.3308	212.118, 165.047, 141.043, 133.088, 89.062	C_27_H_48_O_9_	2.07	MGMG 18:2	1.52	0.60	0.0357	2.00
27.32	[M+H]^+^	760.5857	759.5785	n.d.				0.31	-1.69	0.0208	1.97
27.64	[M-H]^-^	433.2351	434.2424	279.230, 171.006, 152.995, 96.970, 78.959	C_21_H_39_O_7_P	2.24	LysoPA 18:2	3.75	1.91	0.0135	2.04
28.73	[M+Na]^+^	780.5520	757.5636	721.470, 597.475, 575.496, 520.330, 502.314, 500.300, 478.317, 465.228, 184.070, 146.979, 86.096	C_42_H_80_NO_8_P	1.78	PC 34:2; PC (16:0/18:2)	0.15	-2.73	0.0318	2.46

RT, Retention time (minutes); Log2FC, Log2 Fold change between resistant and susceptible RILs; p, p-value for the t-test; V1, VIP score in the OPLS-DA model. n.d. stands for non detected.

**Table 2:** LC-MS/MS data, tentative annotation, fold changes, *p* values and VIP scores for differentially accumulated metabolites between resistant and susceptible maize RILs at 10 days after inoculation with *Fusarium verticillioides*.

RT (min)	Ionization	m/z	Neutral mass	MS/MS fragments (m/z)	Molecular formula	Error (|Δ m/z| ppm)	Tentative compound	FC	log_2_FC	*p*	FDR	VIP
0.75	[M+H]^+^	146.1652	145.1579	n.d.	C_7_H_19_N_3_	0.24	Spermidine	1.84	0.88	0.0001	0.05	2.87
0.91	[M+H]^+^	222.9881	221.9809	n.d.				1.66	0.74	0.0215	0.32	1.88
0.92	[M+H]^+^	185.0327	184.0255	124.997, 116.993, 98.982, 86.095, 71.072, 45.033				1.64	0.71	0.0422	0.4	1.62
0.92	[M-H]^-^	298.1141	299.1214	132.030, 118.050, 96.970, 74.027, 72.010				1.71	0.77	0.0190	0.31	1.97
0.94	[M+H]^+^	351.069	350.0618	n.d.				2.48	1.31	0.0020	0.08	2.47
1.05	[M+H]^+^	179.0485	178.0413	n.d.	C_5_H_10_N_2_O_3_S	0.18	Cysteinylglycine	1.81	0.86	0.0153	0.31	2.05
1.05	[M+H]^+^	308.0922	307.0849	142.031, 116.016, 104.070, 84.045, 76.022	C_10_H_17_N_3_O_6_S	3.59	Glutathione	1.79	0.84	0.0099	0.27	2.15
1.07	[M+H]^+^	492.1814	491.1742	n.d.				1.87	0.91	0.0124	0.27	2.08
1.08	[M+H]^+^	150.0585	149.0513	84.044, 65.039, 61.010, 56.048	C_5_H_11_NO_2_S	0.83	Methionine	1.82	0.86	0.0260	0.34	1.83
1.49	[M+H]^+^	308.0908	307.0836	142.031, 116.016, 104.070, 84.045, 76.022	C_10_H_17_N_3_O_6_S	0.87	Glutathione	1.61	0.68	0.0235	0.32	1.94
1.50	[M+H]^+^	179.0483	178.0410	n.d.	C_5_H_10_N_2_O_3_S	0.97	Cysteinylglycine	1.58	0.66	0.0223	0.32	1.96
1.50	[M+H]^+^	102.055	101.0492	n.d.	C_4_H_7_NO_2_	1.3	Methionine derivative	1.56	0.64	0.0061	0.2	2.22
1.50	[M+H]^+^	133.0319	132.0247	108.955, 90.954, 87.022, 72.004, 61.013, 56.051	C_5_H_8_O_2_S	1.46	Methionine derivative	1.52	0.60	0.0240	0.32	1.91
1.50	[M+H]^+^	150.0583	149.0511	84.044, 65.039, 61.010, 56.048	C_5_H_11_NO_2_S	0.14	Methionine	1.64	0.71	0.0127	0.27	2.05
9.35	[M+H]^+^	130.0648	129.0576	n.d.	C_9_H_7_N	2.17	Isoquinoline	1.70	0.77	0.0130	0.27	2.16
9.35	[M+Na]^+^	360.1056	337.1164	306.061, 259.688, 167.054, 131.066, 130.062, 94.035, 57.070	C_16_H_19_NO_7_	1.02	Indole-3-acetyl-hexoside	1.69	0.76	0.0126	0.27	2.16
10.82	[M+HCOOH-H]^-^	544.1667	499.1684	498.154, 341.111, 323.099, 245.070, 203.057, 179.058, 174.057, 161.047, 143.036, 130.067, 119.036, 101.024, 89.026	C_22_H_29_NO_12_	0.60	Indole-3-acetyl-*myo*-inositol hexoside	2.46	1.30	0.0011	0.07	2.55
10.83	[M+Na]+	522.1581	499.1689	499.125, 360.100, 203.358, 176.069, 130.062	C_22_H_29_NO_12_	0.42	Indole-3-acetyl-*myo*-inositol hexoside	2.80	1.48	0.0005	0.07	2.72
10.83	[M+H]^+^	338.1231	337.1162	n.d.	C_16_H_19_NO_7_	0.09	Indole-3-acetyl-hexoside	2.80	1.49	0.0007	0.07	2.64
10.86	[M+H]^+^	130.065	129.0577	118.064, 115.017, 105.045, 95.055, 90.947	C_9_H_7_N	0.94	Isoquinoline	2.80	1.49	0.0010	0.07	2.60
10.89	[M+Na]^+^	360.1056	337.1164	190.063, 169.027, 167.050, 138.953, 130.064	C_16_H_19_NO_7_	1.17	Indole-3-acetyl-hexoside	2.03	1.02	0.0066	0.2	2.27
12.59	[M-H_2_O+H]^+^	207.065	224.0683	149.019, 135.040, 119.048, 105.044, 91.054, 65.040, 53.039	C_11_H_12_O5	0.73	Sinapic acid	1.95	0.97	0.0163	0.31	1.93
24.01	[M+H]^+^	337.2732	336.2659	335.143, 296.288, 195.085, 188.089, 119.058, 109.100, 95.083, 81.069	C_21_H_36_O_3_	1.37	Glycidyl linoleate	2.05	1.03	0.0181	0.31	1.82
24.01	[M-H]^-^	476.2763	477.2836	279.233, 214.049, 196.038, 140.012, 78.690	C_23_H_44_NO_7_P	4.30	LysoPE 18:2	1.77	0.82	0.0389	0.39	1.54
24.01	[M+H]^+^	478.2934	477.2863	337.268, 306.271, 263.228, 173.015, 109.099, 95.083, 81.069, 62.062	C_23_H_44_NO_7_P	4.30	LysoPE 18:2	2.09	1.06	0.0191	0.31	1.81
24.09	[M+H]^+^	184.0737	183.0665	n.d.	C_5_H_14_NO_4_P	2.41	Choline phosphate	1.65	0.72	0.0435	0.4	1.57
24.09	[M+H]^+^	520.3404	519.3331	502.320, 377.268, 258.104, 184.065, 124.996, 104.105, 86.095	C_26_H_50_NO_7_P	1.11	LysoPC 18:2	1.67	0.74	0.0340	0.38	1.66
24.09	[2M+H]^+^	1039.674	519.3337	n.d.	C_26_H_50_NO_7_P	2.48	LysoPC 18:2	2.89	1.53	0.0495	0.43	1.49
25.01	[M+H]^+^	337.2736	336.2664	335.143, 296.288, 195.085, 188.089, 119.058, 109.100, 95.083, 81.069	C_21_H_36_O_3_	0.20	Glycidyl linoleate	1.86	0.89	0.0172	0.31	1.89
25.16	[M+Na]^+^	539.32	516.3306	486.279, 402.982, 328.154, 264.243, 230, 200, 163, 124, 104, 87, 57	C_27_H_48_O_9_	1.81	MGMG 18:2	1.61	0.69	0.0437	0.4	1.57
25.16	[M+HCOOH-H]^-^	561.3261	516.3279	506.324, 281.249, 279.233, 253.093, 224.070, 44.998	C_27_H_48_O_9_	3.85	MGMG 18:2	1.64	0.71	0.0223	0.32	1.75
27.57	[M+H]^+^	441.1595	440.1523	n.d.				2.78	1.48	0.0305	0.37	1.71
27.58	[M+H]^+^	119.0854	118.0782	n.d.				2.21	1.15	0.0409	0.4	1.62
27.58	[M+H]^+^	147.081	146.0737	103.052, 91.052, 62.926				2.77	1.47	0.0348	0.38	1.67
27.58	[M+H]^+^	559.284	558.2767	n.d.				2.75	1.46	0.0238	0.32	1.77
27.64	[M-H]^-^	433.2342	434.2415	431.193, 279.236, 152.996, 96.970, 78.959	C_21_H_39_O_7_P	4.35	LysoPA 18:2	16.70	4.06	0.0067	0.2	2.07
28.69	[M-H]^-^	379.157	380.1643	367.157, 349.148, 279.230, 116.925, 99.926, 84.950, 44.998			Octadecadienoic acid derivative	0.57	-0.81	0.0013	0.07	2.54
28.70	[M+Na]^+^	804.5536	781.5643	745.458, 621.469, 599.489, 146.978, 89.094	C_44_H_80_NO_8_P	2.84	PC 36:4; PC (18:2/18:2)	2.21	1.14	0.0358	0.38	1.67
28.81	[M+HCOOH-H]^-^	826.5574	781.5592	766.538, 504.307, 486.302, 430.272, 279.232, 224.068, 168.044, 44.996	C_44_H_80_NO_8_P	3.19	PC 36:4; PC (18:2/18:2)	3.35	1.75	0.0003	0.07	2.72
29.33	[M+HCOOH-H]^-^	802.5574	757.5592	742.540, 504.307, 486.296, 480.309, 462.299, 279.233, 255.233, 224.069, 168.042, 44.997	C_42_H_80_NO_8_P	3.83	PC 34:2; PC (16:0/18:2)	2.44	1.28	0.0007	0.07	2.75
29.64	[M-H]^-^	279.1621	280.1694	200.465, 155.081, 133.141, 96.960, 79.956	.			0.34	-1.55	0.0016	0.07	2.47

RT, Retention time (minutes); Log_2_FC, Log_2_ Fold change between resistant and susceptible RILs; p, p-value for the t-test; FDR, p-adjusted value; VIP, VIP 1 score in the OPLS-DA model. n.d. stands for non detected.

When possible, the differentially accumulated metabolite features in kernels were putatively annotated based on public databases and literature references, and about 75% of them could be related to known compounds (**Tables 1**, **2**). A total of 30% were lipid or lipid-related compounds, 21% were related to methionine and glutathione, 20% were indole-3-acetic acid (IAA) derivative compounds, and only 3% were phenolic compounds. Compounds displayed a deprotonated ion at m/z 337 were annotated as IIA-hexose based on the exact mass and the characteristic fragmentation ions at m/z 176 (IAA) and at m/z 130 (isoquinoline). The fragment at m/z 176 (IAA) was generated by a neutral loss of 162 Da of one hexose that probably correspond to *myo*-inositol, since the forms of indole-3-acetyl-*myo*-inositol are the most abundant IAA conjugates in maize kernels (Bandurski, 1979; Ostrowski et al, 2020). Thus, ions [M+Na]^+^ at m/z 522 and [M+HCOOH-H]^-^ at m/z 544 were assigned as sodium and formic acid adducts of IAA-*myo*-inositol-hexose (499.1693 Da). Compounds eluted at 1.5 min with [M+H]^+^ ions at m/z 133, 104 and 102 were assigned as dissociation products of methionine (m/z 150) eluted at the same retention time (Kotiaho et al, 2000). Compound with an [M-H]^-^ ion at m/z 164 in 3 dat samples was annotated as the benzoxazinoid 6-methoxy-2-benzoxazolinone (MBOA) based on its fragmentation pattern at m/z 164, 149, 121 according to Bruijn et al. (2016). Metabolite displayed a [M-H]^-^ ion at m/z 379 at 28.6 min in 10 dat samples was left as unidentified. However, the fragment ion at m/z 279.23 and the detection of a octadecadienoic acid form ([M-H]^-^ at m/z 279.23) at the same retention time, suggested that it could be a fatty acid related compound.

**Figure 3:**
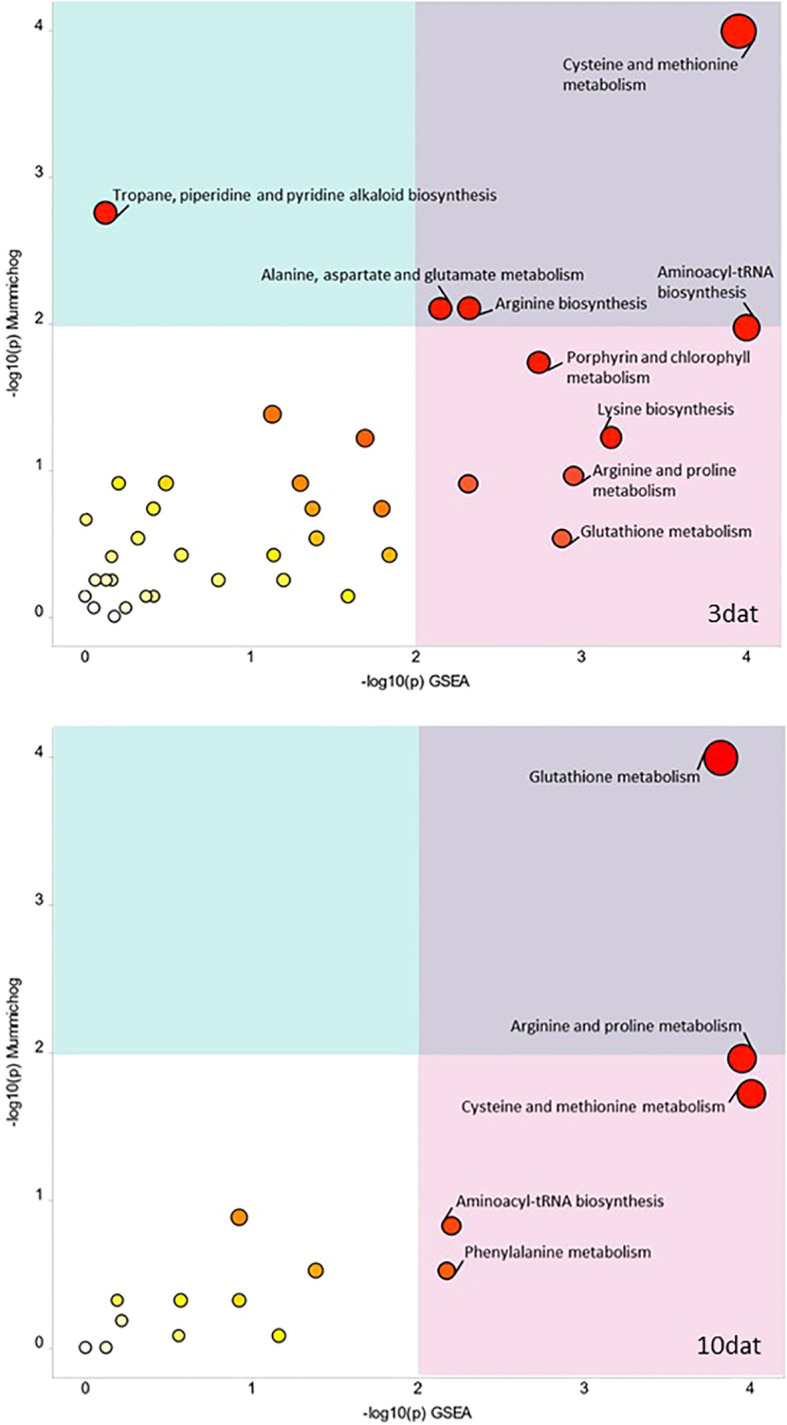
Functional analysis using the Oryza database of metabolomic features detected in maize kernels collected 3 (3dat) and 10 (10dat) days after inoculation (dat) with F. verticillioides in resistant and susceptible RILs. This analysis uses the “mummichog” algorithm to predict pathway activities based on a list of peaks ranked based on t-tests (Pang et al., 2021). Larger and more reddish circles (smaller combined p-value) represent more reliably perturbed pathways.

The Functional Analysis results showed several pathways such as the cysteine and methionine metabolism; aminoacyl-tRNA biosynthesis; arginine biosynthesis; alanine, aspartate and glutamate metabolism; and porphyrin and chlorophyll metabolism that were significantly (combined *p*-values < 0.01) enriched among metabolites differentially accumulated in 3 dat kernel samples of resistant and susceptible RILs (**Figure 3**). Meanwhile, the pathway enrichment analysis for metabolites in kernel samples collected at 10 dat showed that the pathways glutathione metabolism; arginine and proline metabolism; and cysteine and methionine metabolism were significantly enriched (**Figure 3**).

Visualization of genes and metabolites differentially regulated in resistant compared to susceptible RILs within three different pathways, phenylpropanoid biosynthesis and glutathione and glycerophospholipid metabolisms can be made through **Supplemental Figures 2**–**4**, respectively.

## Discussion

### Higher metabolic differences at later stage of Fusarium infection

The metabolic profiles in maize kernels from RILs with contrasting resistance-susceptibility levels to *F. verticillioides* infection and fumonisin accumulation were explored at 3 and 10 days after infection with the fungus. Results of the supervised OPLS-DA show that differences between resistant and susceptible RILs were better explained by metabolite abundances at 10 dat than at 3 dat. In addition, high-significant differentially accumulated metabolites were only found at 10 dat. This suggests a larger response occurs at later stages of *F. verticillioides* infection of maize kernels. These results agree with those obtained by Wang et al. (2016) comparing gene expressions at 1 and 10 dat; these authors suggested that genes involved in biosynthesis of secondary metabolites with antifungal effects would be preferentially induced at the late stage of *F. verticillioides* colonization. Conversely, functional analyses showed that aminoacyl-tRNA biosynthesis and cysteine, methionine, arginine, proline and glutathione metabolic pathways were enriched among metabolites differentially accumulated in resistant and susceptible RILs in both sampling dates. Therefore, we could hypothesize that some final changes could be driven by subtle differences at earlier stages of *Fusarium* infection between resistant and susceptible inbreds. That is the case for isoquinoline, a type of alkaloid with a clear fungicidal effect and proven inhibitory effects on the synthesis of fumonisins by *Fusarium oxysporum* (Tims and Batista, 2007). However, at 10 dat, fewer features differentially accumulated were detected in comparison to differentially transcribed genes (364) between resistant and susceptible RILs that could suggest that a longer period of exposition to the fungus is necessary to reveal all relevant metabolome differences between resistant and susceptible inbreds (Cao et al., 2022). Therefore, metabolome and transcriptome studies would bring complementary results although some pathways detected by both approaches will be emphasized.

### Importance of the membrane lipid homeostasis in resistance

Three probable lysophospholipids were differentially accumulated in resistant vs susceptible RILs at 10 dat, but those changes co-occurred with a more significant difference for phosphatidylcholines content between resistant and susceptible RILs. Similarly, an untargeted approach to look for metabolites involved in kernel resistance to fumonisin contamination showed that kernel lipid signature at harvest was strongly involved in the plant−pathogen interaction and in the modulation of fumonisin accumulation; the phosphatidylcholine PC(O-16:0/18:2) being significantly more accumulated in the resistant than in the susceptible hybrid at harvest (Righetti et al, 2019; Righetti et al, 2021). The gene Zm00001d010840, that encodes the enzyme triacylglycerol lipase-like 1, has been proposed as QTL candidate gene for fumonisin content since this gene was significantly upregulated in the resistant RILs compared to the susceptible ones, and was within the confidence interval of one QTL for fumonisin content detected in the RIL population from which the eight RILs used in the current study were selected (Cao et al, 2022). This gene could be involved in degradation of oil bodies in seeds by hydrolysis of ester linkages of triglycerides to diacylglycerol which can be re-directed to the synthesis of lysophospholipids and, specially, phosphatydilcholine in the resistant RILs (Dubots et al, 2012; Wang et al, 2012; Lu et al, 2020; Bates, 2022). The higher accumulation of phosphatidylcholines, key building blocks of membrane bilayers, in resistant RILs could contribute to ROS scavenging as it has been shown that phosphatidylcholine inhibits lipid oxidation synergistically with primary antioxidants, especially tocopherols (Cui and Decker, 2016). In addition, exogenous application of phosphatidylcholine had a positive effect on maintaining cell integrity and cell-membrane structure possibly through keeping cell membrane phospholipid homeostasis (Sun et al, 2022). On the other hand, head-group acylation of mono- and digalactosyldiacylglycerols (MGDC and DGDG, respectively), main lipids in plastid membranes (amyloblasts, seed plastids in which starch is synthesized and stored, membranes contain MGDG and DGDG in high abundance), is a common stress response in plants. In this sense, the galactolipid monogalactosylmonoacylglycerol (18:2) (MGMG) derived from the acylation of MGDG could contribute to the maintenance of galactolipid homeostasis (Myers et al, 2011; Shimojima and Ohta, 2022). Therefore, lipid differences found between resistant and susceptible RILs could be a key factor in order to increase the stability of cell membranes during *F. verticillioides* infection.

### The multifunctional role of methionine metabolism in resistance

The amino acid methionine is the immediate precursor of *S*-adenosylmethionine (SAM), the major methyl-group donor in transmethylation reactions and intermediate in the biosynthesis of compounds related to plant defense against pathogens such as glutathione, polyamines and the phytohormone ethylene (Ravanel et al, 1998; Nikiforova et al, 2002; Makinen and De, 2019). Methyl groups of choline, phosphatidylcholine, and phosphorylcholine being major end products of transmethylation by SAM (Giovanelli et al, 1985). In kernels of the resistant RILs collected at 10 dat, Cao et al. (2022) already showed that genes involved in ethylene signaling were upregulated compared with the susceptible ones. In the current study, glutathione and spermidine, a polyamine that at a concentration of 1 ng/mL has proven to inhibit *in vitro* fumonisin production in an 80% with no effect on *F. proliferatum* growth, were more accumulated in the kernels of resistant RILs. These results suggested that increased levels of methionine at earlier stages of fungal infection could lead to increased accumulation of metabolites involved in detoxification and inhibition of fumonisins (Maschietto et al, 2016; Perincherry et al, 2021). Higher contents of spermidine and/or spermine have been also associated to higher resistance to *Aspegillus flavus* and aflatoxin contamination (Majumdar et al, 2019). Kovács et al. (2023) showed that, after inoculation with *Fusarium verticillioides*, seedlings of a tolerant inbred line presented higher levels of spermidine in the radicle than those of a susceptible inbred although differences were not significant. The visual integration of transcriptomic and metabolomic data showed that genes involved in glutathione catabolism were inhibited meanwhile spermidine and reduced glutathione increased in resistant *versus* susceptible RILs (**Supplemental Figure 1**). Therefore, we hypothesize that interplay between spermidine and ROS homeostasis could have and important role in controlling fumonisin accumulation in a similar mechanism to that showed by the plant to control Na^+^ toxicity (Saha et al, 2015; Chen et al, 2017). Chen et al. (2017) showed that spermidine was a key molecule for inducing plant salt tolerance through accumulation of glutathione to reduce damage by reactive oxygen species and regulation of the salt overly sensitive pathway that led to detoxification of Na^+^. Saha et al. (2015) suggested that polyamines modulate ROS homeostasis by the shift between polyamine anabolism and catabolism resulting in a lower polyamine concentration which, in turn, may favor programmed cell death (Saha et al, 2015). According with this hypothesis, Cao et al. (2022) found that genes involved in programmed cell death were downregulated in the resistant RILs compared to the susceptible ones and the candidate gene proposed (probably encoding an NF-κB inhibitor-like protein) for the most relevant QTL for fumonisin content could participate in preventing polyamide catabolism (Liu et al, 2020).

### Modulation of IAA conjugates in relation to resistance

IAA-*myo*-inositol and its glycosidic forms with galactose and arabinose are the major IAA ester conjugates in maize seed endosperm; IAA ester conjugates comprising the 97-99% of endogenous IAA (Ostrowski et al, 2020). Cao et al. (2022) found that genes involved in auxin signaling repression were upregulated in the resistant compared to susceptible RILs using the same kernel samples, agreeing with the idea that induced auxin biosynthesis or modulated auxin signaling is associated with increased host susceptibility (Brauer et al, 2019). The net level of free IAA in the cell is determined by hormone IAA synthesis, conjugation, degradation and transport, but *Fusarium* species appear much more likely to manipulate plant auxin homeostasis by hydrolysis of IAA-amino acid and sugar conjugates or perhaps, regulating enzymes participating in synthesis, than by *de novo* synthesis (Vrabka et al, 2019). Therefore, the higher accumulation of IAA-conjugates, particularly IAA-*myo*-inositol conjugates (immature maize seeds do not produce amide linkages of IAA), in resistant compared to susceptible RILs could be related to reduced susceptibility by controlling auxin homeostasis (Ostrowski et al, 2020).

### Other metabolites likely related to resistance

Therefore, subtle changes observed at 3 dat for isoquinoline and metabolites involved in methionine metabolism, lipid remodeling, IAA signaling are kept at 10 dat and, in some cases, amplified as it is the case for phosphatidylcholines, isoquinoline and IAA-conjugates. However, there were other metabolites that were high-differentially accumulated in kernel collected at 10 dat but they did not appear as differentially accumulated at 3 dat: the polyamine spermidine, already discussed, and an octadecadienoic acid derivative were over and under-accumulated, respectively, in resistant compared to susceptible RILs. Octadecadienoic acid derivatives such as the Lox3 derived oxylipins haves been already associated to increased fumonisin content (Dall’Asta et al, 2012; Battilani et al, 2018; Righetti et al, 2019). Sinapic acid also increased in resistant *versus* susceptible RILs at 10 dat and deserves to be discussed as it has been found along with two other compounds as the only discriminants between phenolic extracts from different parts of the same mushroom species (*Lentinula edodes*) with very contrasting effects on fumonisin biosynthesis and no effect on *F. verticillioides* biomass (Merel et al, 2020).

Finally, ferulic acid and the benzoxacinoid MBOA appeared as discriminant between resistant and susceptible RILs at 3 dat, and no longer, but they deserve special attention because these metabolites have been described as involved in resistance to fungal diseases but in the current study were more accumulated in the susceptible RILs. Benzoxazinoids are abundant indole-derived specialized metabolites in several monocot crop species that function as iron chelators, allelochemicals and in plant defense against herbivorous arthropods and fungal pathogens (Niemeyer, 2009 **;** Poschenrieder et al, 2005 **).** Benzoxazinoid hydroxamic acids are stored as inactive glucosides in the vacuoles, but they are enzymatically converted to the active aglycones by glucosidases upon plant cell disruption and the resulting aglycones are further degraded spontaneously to the corresponding benzoxazolinones, MBOA and its desmethoxy derivative (BOA) (Hashimoto and Shudo, 1996). Therefore, higher accumulation of MBOA could be associated to increased cell damage which promotes H_2_O_2_ and, consequently, ferulic acid accumulation, but would be only evident at early stages of infection because *F. verticillioides* can metabolize and detoxify active benzoxazinoids such as MBOA (Glenn et al, 2001; Jabeen et al, 2007; Li et al, 2020). According to the hypothesis of increased cell damage in susceptible inbreds, in a previous transcriptomic study with the same materials, genes related to cell death were downregulated in the resistant compared with the susceptible RILs (Cao et al, 2022).

Metabolomic differences between resistant and susceptible RILs would confirm some results from the previous transcriptomic study (Cao et al, 2022) such as the down-regulation of auxin signaling, up-regulation of the phenylpropanoid pathway, activation of electron transport chain toward amino acid synthesis and reduced oxidative stress in the resistant RILs compared with the susceptible ones. However, in the current study, new insights on the pathways involved in resistance have been uncovered because the specific increase in methionine metabolism toward polyamine and glutathione over accumulation in resistant versus susceptible RILs has been revealed. Similarly, Cao et al. (2022) suggested that mobilization of lipids from oil bodies to phytoalexin synthesis could have an important role in resistance, but the metabolic study have shown that lipid re-modelling would be rather re-directed toward phosphatidylcholine accumulation.

## Conclusions

Discriminant metabolites between resistant and susceptible RILs were rather found at 10 than 3 dat, although differences for key metabolites were kept across kernel sampling times suggesting that longer exposition to fungal colonization is necessary to uncover biomarkers but those changes could be driven by subtle changes at earlier stages of infection. Within this context, differences for membrane lipid homeostasis, methionine metabolism and IAA-conjugation seemed highly relevant in order to distinguish between resistant and susceptible inbreds. In addition, some metabolites such as spermidine and isoquinoline seemed to be promising indirect traits to improve resistance to FER and reduce fumonisin accumulation. However, their role in resistance should be validated using *in vitro* and *in vivo* experiments to determine the real effects of these compounds on *F. verticilllioides* growth and fumonisins biosynthesis.

## Data availability statement

Raw metabolic data are deposited in the online repository DIGITAL.CSIC and can be accessed using this link: https://doi.org/10.20350/digitalCSIC/15427.

## Author contributions

AB conceived the study. AC took care of field experiments, data recording, and sample collection with the assistance of AB, RS, and RM. AC led extraction and acquisition and filtering of UHPLC-QTOF data with the assistance of NG. AC performed the identification of tentative compounds. AB performed statistical analyses of data. AB and NG drafted the initial manuscript and RS and NG edited the figures. All authors contributed to the article and approved the submitted version.

## Supplementary material

Supplementary Figure 1 Validation of supervised least square and orthogonal projections to latent structures discriminant analysis (OPLS-DA) for data acquired at 3 and 10 days after inoculation (dat) with *Fusarium verticillioides* in resistant and susceptible RILs. **(A)** Cross-validation for R2X, R2Y, and Q2 coefficients for the OPLS-DA model with one predictive (p1) and three orthogonal (o1, o2, and o3) components for samples collected at 3 dat, **(B)** Visualization of permutation test to validate the results of OPLS-DA analysis for samples collected at 3 dat, **(C)** Cross-validation for R2X, R2Y, and Q2 coefficients of the OPLS-DA model with one predictive (p1) and three orthogonal (o1, o2, and o3) components for samples collected at 10 dat, and **(D)** Visualization of permutation test to validate the results of OPLS-DA analysis for samples collected at 10 dat.

Supplementary Figure 2 Integrative visualization by Pathview platform of genes (Cao et al, 2022) and metabolites of the phenylpropanoid biosynthesis pathway differentially expressed and accumulated, respectively, in immature maize kernels of resistant and susceptible RILs to FER and fumonisin contamination collected 10 days after inoculation with *F. verticillioides*.

Supplementary Figure 3 Integrative visualization by Pathview platform of genes (Cao et al, 2022) and metabolites of the glutathione metabolism pathway differentially expressed and accumulated, respectively, in immature maize kernels of resistant and susceptible RILs to FER and fumonisin contamination collected 10 days after inoculation with *Fusarium verticillioides*.

Supplementary Figure 4 Integrative visualization by Pathview platform of genes (Cao et al, 2022) and metabolites of the glycerophospholipid metabolism pathway differentially expressed and accumulated, respectively, in immature maize kernels of resistant and susceptible RILs to FER and fumonisin contamination collected 10 days after inoculation with *Fusarium verticillioides*.
