# Supplementary figures and images for: Maize kernel metabolome involved in resistance to fusarium ear rot and fumonisin contamination
Source: Front Plant Sci. 2023 Jul 19;14:1160092. doi: 10.3389/fpls.2023.1160092 (PMC10394704; doi:10.3389/fpls.2023.1160092)

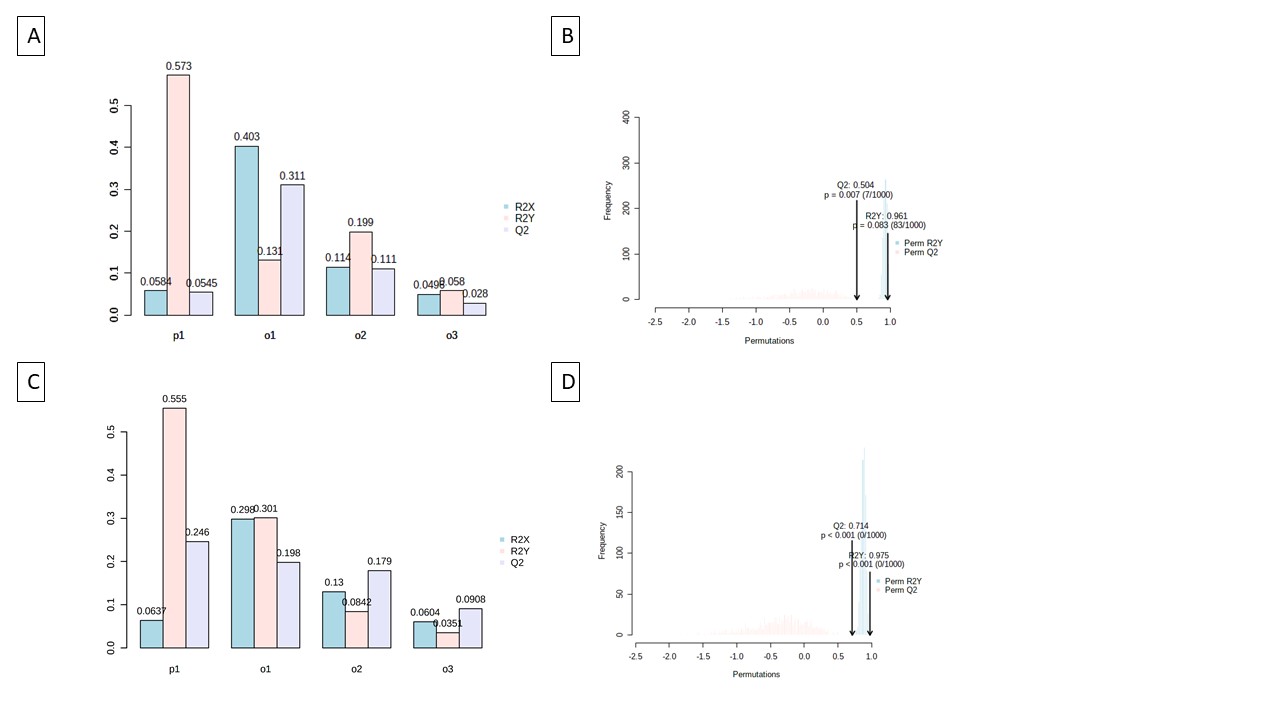

Supplement: Supplementary Figure 1 — Validation of supervised least square and orthogonal projections to latent structures discriminant analysis (OPLS-DA) for data acquired at 3 and 10 days after inoculation (dat) with Fusarium verticillioides in resistant and susceptible RILs. (A) Cross-validation for R2X, R2Y, and Q2 coefficients for the OPLS-DA model with one predictive (p1) and three orthogonal (o1, o2, and o3) components for samples collected at 3 dat, (B) Visualization of permutation test to validate the results of OPLS-DA analysis for samples collected at 3 dat, (C) Cross-validation for R2X, R2Y, and Q2 coefficients of the OPLS-DA model with one predictive (p1) and three orthogonal (o1, o2, and o3) components for samples collected at 10 dat, and (D) Visualization of permutation test to validate the results of OPLS-DA analysis for samples collected at 10 dat. [file Image_1.jpeg]

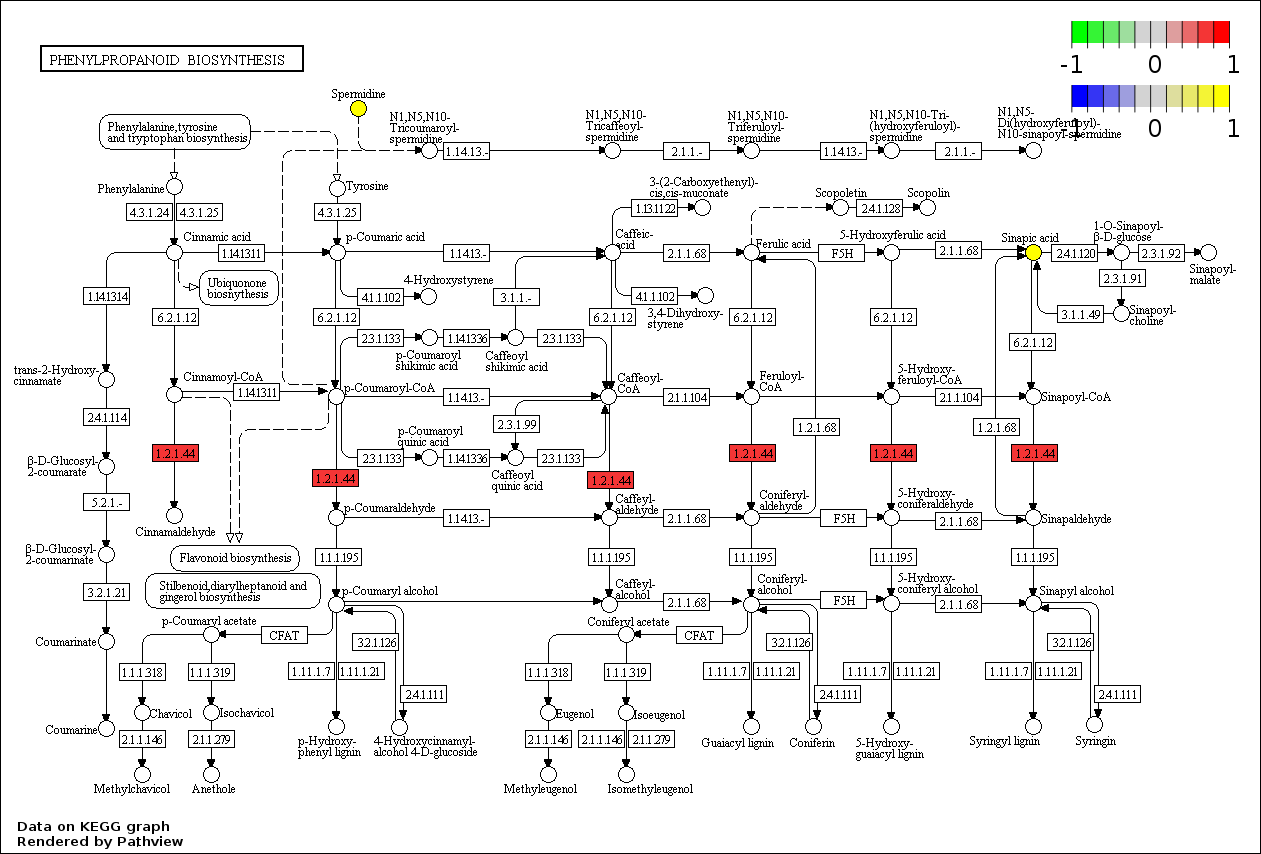

Supplement: Supplementary Figure 2 — Integrative visualization by Pathview platform of genes (Cao et al, 2022) and metabolites of the phenylpropanoid biosynthesis pathway differentially expressed and accumulated, respectively, in immature maize kernels of resistant and susceptible RILs to FER and fumonisin contamination collected 10 days after inoculation with F. verticillioides. [file Image_2.png]

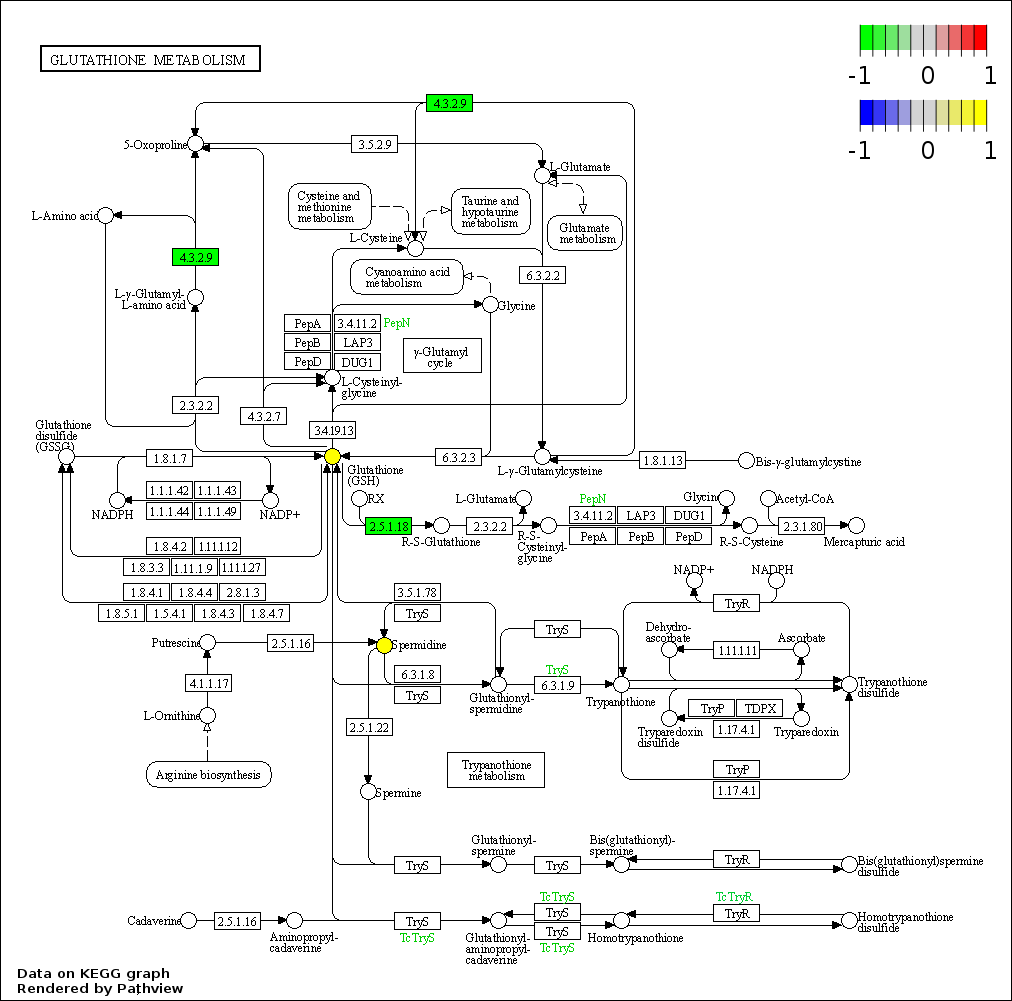

Supplement: Supplementary Figure 3 — Integrative visualization by Pathview platform of genes (Cao et al, 2022) and metabolites of the glutathione metabolism pathway differentially expressed and accumulated, respectively, in immature maize kernels of resistant and susceptible RILs to FER and fumonisin contamination collected 10 days after inoculation with Fusarium verticillioides. [file Image_3.png]

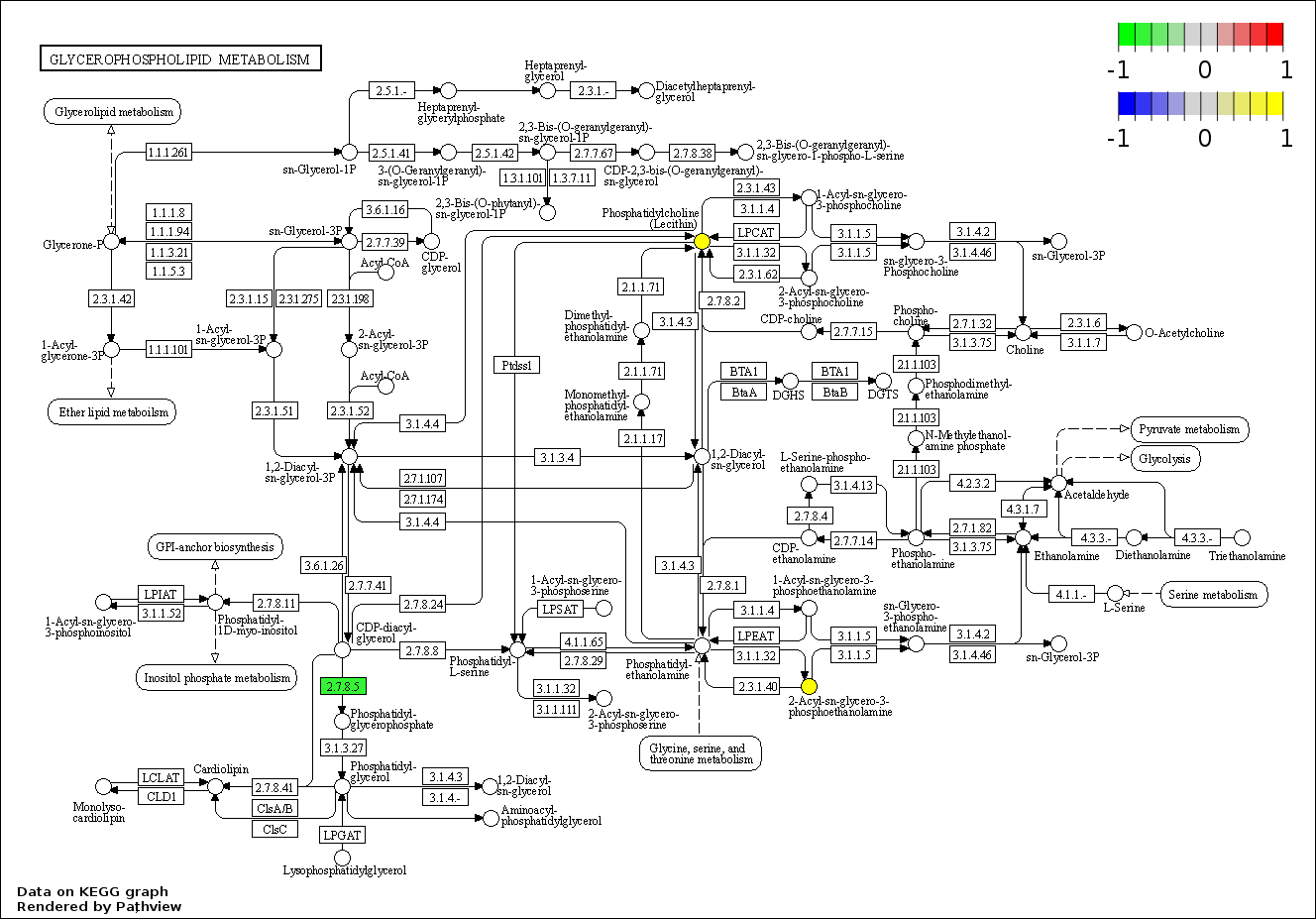

Supplement: Supplementary Figure 4 — Integrative visualization by Pathview platform of genes (Cao et al, 2022) and metabolites of the glycerophospholipid metabolism pathway differentially expressed and accumulated, respectively, in immature maize kernels of resistant and susceptible RILs to FER and fumonisin contamination collected 10 days after inoculation with Fusarium verticillioides. [file Image_4.png]
