# Supplementary material for: Maize kernel metabolome involved in resistance to fusarium ear rot and fumonisin contamination
Source: Front Plant Sci. 2023 Jul 19;14:1160092. doi: 10.3389/fpls.2023.1160092 (PMC10394704; doi:10.3389/fpls.2023.1160092)
Supplement: Supplementary file 5 [file Table_1.docx]

Supplementary table 1. Recombinant inbred lines (RIL) used in the metabolomic study classified as resistant or susceptible to Fusarium ear rot and kernel contamination with fumonisins on the basis of a previous evaluation (Cao et al. 2022)

| RIL | Group | Fusarium ear rot | Fumonisin content (mg/kg) |
| --- | --- | --- | --- |
| RIL125 | Resistant | 2.3 | 11.2 |
| RIL128 | Resistant | 2.2 | 11.6 |
| RIL114 | Resistant | 2.6 | 13 |
| RIL279 | Resistant | 2.5 | 13.3 |
| RIL14 | Susceptible | 3.8 | 76 |
| RIL348 | Susceptible | 4.1 | 62.7 |
| RIL319 | Susceptible | 3.7 | 55.3 |
| RIL152 | Susceptible | 4.2 | 55.3 |

Averaged data of recombinant inbred lines (RIL) for Fusarium ear rot (on a 7-point visual scale: 1 = no visible disease symptoms; 2 = 1 to 3 %; 3 = 4 to 10 %; 4 = 11 to 25 %; 5 = 26 to 50 %; 6 = 51 to 75 % and 7 = 76 to 100% of kernels exhibiting visual symptoms of infection) and fumonisin content have been taken from Cao et al. (2022).
